# Fenofibrate suppresses *Mycoplasma bovis* infection via autophagy-mediated cholesterol regulation in bovine mammary epithelial cells and murine mammary tissue

**DOI:** 10.3389/fcimb.2025.1731492

**Published:** 2026-01-12

**Authors:** Maolin Xu, Tian Wang, Xian Deng, Yuxin Liu, Zimeng Zhu, Herman W. Barkema, Eduardo R. Cobo, John P. Kastelic, Xueying Zhou, Bo Han

**Affiliations:** 1 College of Veterinary Medicine, China Agricultural University, Beijing, China; 2 Faculty of Veterinary Medicine, University of Calgary, Calgary, AB, Canada

**Keywords:** autophagy, cholesterol metabolism, fenofibrate, intracellular infection, *Mycoplasma bovis*

## Abstract

**Background:**

*Mycoplasma bovis* mastitis is an important disease of dairy cows; however, there are no commercial *M. bovis* vaccines and antimicrobial resistance is increasing. Furthermore, *M. bovis* lacks a cell wall and relies on host-derived cholesterol for survival and growth.

**Methods:**

We evaluated effects of fenofibrate, a peroxisome proliferator-activated receptor α (PPARα) agonist, on *M. bovis* infection, using both bovine mammary epithelial cells and a murine mastitis model. *In vitro* analyses assessed autophagy, nuclear Transcription Factor EB (TFEB) and Transcription Factor E3 (TFE3) translocation, cholesterol metabolism, and bacterial localization, whereas *in vivo* evaluations included inflammatory responses, lysosomal/autophagy protein expression, cholesterol content, and bacterial burden.

**Results:**

Fenofibrate enhanced autophagic flux via upregulation of LC3B and LAMP2, promoted nuclear translocation of TFEB and TFE3, and reduced intracellular cholesterol by repressing 3-Hydroxy-3-Methylglutaryl-Coenzyme A Reductase (HMGCR) and Sterol Regulatory Element-Binding Transcription Factor 2 (SREBF2) while increasing Acetyl-Coenzyme A Acetyltransferase 1 (ACAT1) expression. Based on confocal imaging, fenofibrate disrupted co-localization between *M. bovis* and free cholesterol while enhancing its uptake by autophagosomes. In a murine mastitis model, fenofibrate alleviated inflammatory cell infiltration and cytokine release, restored lysosomal and autophagy protein expression, reduced cholesterol content, and significantly lowered bacterial burden.

**Conclusion:**

Fenofibrate enhanced defense capability of mammary epithelial cells against *M. bovis* infection through a dual mechanism—promoting autophagy and regulating cholesterol homeostasis—thereby reducing bacterial survival and protecting tissues from damage. This discovery provides a novel strategy for prevention and treatment of *M. bovis* infection, warranting further investigation in bovine models to assess pharmacokinetics, dosage, and clinical efficacy.

## Introduction

*Mycoplasma bovis* (*M. bovis*) is an important pathogenic bacterium responsible for chronic respiratory disease, mastitis, arthritis, and otitis in cattle, leading to substantial economic losses in the global livestock industry (Gelgie et al., 2022; Okella et al., 2023; Dudley et al., 2025). With no cell wall, *M. bovis* has intrinsic resistance to antibiotics targeting cell wall biosynthesis, e.g., penicillins and cephalosporins (Gautier-Bouchardon, 2018). Moreover, its cholesterol-rich plasma membrane and inability to synthesize cholesterol *de novo* render it fully dependent on host-derived sterols. As *M. bovis* needs host cholesterol to preserve membrane integrity and support intracellular proliferation (Adamu et al., 2020), cholesterol metabolism is a promising target for therapeutic intervention.

Cellular invasion is a critical strategy for pathogens to establish persistent infections and evade immune clearance (Askar et al., 2021; Li et al., 2024a). *M. bovis* is a facultative intracellular pathogen, capable of invading and colonizing host cells, contributing to its persistence (Zhao et al., 2022). With rising antimicrobial resistance and no effective vaccines, there is an urgent need to develop approaches that target host-pathogen interactions rather than the pathogen (Li et al., 2024b). Emerging research highlights 2 critical host cellular processes that influence *M. bovis* infection: cholesterol metabolism and autophagy. Cholesterol homeostasis is a critical pathogenic regulatory target for various intracellular invasive bacteria, including *Mycobacterium tuberculosis*, *Brucella* spp., *Salmonella* spp (Cian et al., 2022), and *Shigella* spp (Rossi et al., 2017). *Mycoplasma* spp., with their remarkably reduced genomes (0.58 to 2.20 Mb, versus 4.64 Mb in *Escherichia coli*), lack genetic machinery for *de novo* lipid biosynthesis. Consequently, their survival and replication are highly dependent on exogenous fatty acids, cholesterol, and complex lipids from the host or culture media (Adamu et al., 2020). Among prokaryotes capable of incorporating cholesterol, *M. bovis* and related species are particularly reliant on it for membrane integrity and pathogenesis. Notably, *Mycoplasma pneumoniae* persists long-term within cholesterol-rich atherosclerotic plaques (Shen et al., 2024). Therefore, disruption of host cholesterol homeostasis has potential to inhibit intracellular proliferation and persistent infection of *M. bovis*.

Autophagy is a fundamental defense mechanism in eukaryotic cells against intracellular pathogens, with a pivotal role in maintaining lipid homeostasis, an essential prerequisite for cellular and systemic physiological integrity (Park et al., 2023; Jarocki et al., 2024). Cholesterol contributes to structural integrity of microdomains within the lysosomal membrane, critical for regulating chaperone-mediated autophagy (CMA) and fusion of autophagosomes with lysosomes (Bik et al., 2021). Notably, short-term cholesterol depletion triggers rapid activation of autophagy. Transcription Factor EB (TFEB) and Transcription Factor E3 (TFE3) are master regulators of lysosomal biogenesis and autophagic flux (Raben and Puertollano, 2016). In particular, TFEB orchestrates global transcriptional control of lipid catabolism by activating peroxisome proliferator-activated receptor gamma coactivator 1-alpha (PGC1α) and Peroxisome proliferator-activated receptor α (PPARα) (Zhang et al., 2024). Moreover, autophagy contributes to host defense against *M. bovis* by mediating intracellular degradation of the pathogen, thereby participating in clearance of infection (Liu et al., 2021).

Fenofibrate, a third-generation fibrate derivative, functions as a potent agonist of peroxisome proliferator-activated receptor alpha (PPARα), a nuclear receptor that regulates lipid and energy metabolism. It has been widely used to treat hypertriglyceridemia and mixed dyslipidemia, lowering circulating triglycerides and low-density lipoprotein cholesterol while elevating high-density lipoprotein cholesterol, thereby reducing the risk of atherosclerosis and cardiovascular events (Almasri et al., 2020; Saha et al., 2023). Furthermore, it has been reported to induce autophagy (Qiu et al., 2023; Cetti et al., 2025) and to exert multiple beneficial effects in disease models. In a murine model of *Trypanosoma cruzi* infection, fenofibrate mitigated inflammation, prevented fibrosis, and improved cardiac function (Ruiz Luque et al., 2024). *In vitro*, it reduced SARS-CoV-2 viral load by ~70% in infected Vero cells (Davies et al., 2021). Both *in vitro* and *in vivo*, fenofibrate-induced PPARα activation enhanced immune responses against bacterial challenges (Andersson et al., 2016; Van Wyngene et al., 2020; Guerra et al., 2024). Given its dual role in lipid regulation and immune modulation, plus good safety profile and extensive clinical application, fenofibrate has potential for treatment of *M. bovis*-induced mastitis.

This study used 2 models, an *in vitro* bovine mammary epithelial cells (bMECs) and an *in vivo* murine mammary gland model, to investigate therapeutic potential of fenofibrate in modulating *M. bovis* infection and intracellular replication via the autophagy-cholesterol axis. Our findings provide the first evidence that autophagy-mediated cholesterol metabolism has a pivotal role in host defense against *M. bovis*, offering new mechanistic insights and identifying cholesterol metabolism as a novel target for therapeutic intervention in bovine mastitis.

## Materials and methods

### Chemicals and reagents

Ad-mCherry-GFP-Microtubule-associated protein 1 light chain 3 beta (LC3B) (C3011), Ad-GFP-LC3B (C3006), DiI (1,1’-dioctadecyl-3,3,3’,3’-tetramethylindocarbocyanine perchlorate, C1036), Hanks’ Balanced Salt Solution (with Ca^2+^& Mg^2+^, HBSS, C0219), coverslips (FCGF60), Hoechst 33258 (C1017), and Histone H3 antibody (AF0009) were from Beyotime (Shanghai, China). Fenofibrate (HY-17356), SBE-β-CD (Sulfobutylether-β-Cyclodextrin, HY-17031), DMSO (Dimethyl sulfoxide, HY-Y0320C), and Filipin complex (HY-N6716) were purchased from MedChemExpress (Monmouth Junction, NJ, USA). Anti-LAMP1 antibody (67300-1-Ig), anti- lysosomal-associated membrane protein 2 (LAMP2) antibody (66301-1-Ig), anti-TFEB antibody (13372-1-AP), anti-TFE3 antibody (14480-1-AP), anti-LC3 polyclonal antibody (14600-1-AP), anti-ATG5 antibody (10181-2-AP), anti-RAB7A antibody (55469-1-AP), anti-mouse IgG-horseradish peroxidase (HRP) (SA00001-1), and Goat anti-rabbit IgG (SA00001-2) were all from Proteintech (Chicago, IL, USA). Anti-SQSTM1/p62 antibody (23214), anti-GAPDH antibody (97166) and Anti-LC3B (E5Q2K) antibody (83506) were from Cell Signaling Technology (Danvers, MA, USA).

### Bacterial strain and cell culture

Two strains of *M. bovis*, PG45 (ATCC 25523) and WT21 (Wild type isolate) were cultured in PPLO medium (BD Biosciences) and yeast extract (BD Biosciences) broth with 20% horse serum (Solarbio, Beijing, China) and 100 IU/L penicillin (Coolaber) in 5% CO_2_ at 37°C for 72 h. The culture was centrifuged (6000 × g for 30 min) and washed with phosphate-buffered saline (PBS). Bovine mammary epithelial cells (bMECs) (MAC-T; Jingma Biological Technology Co., Ltd., Shanghai, China) were cultured in DMEM (Gibco™, 11995073) supplemented with 10% fetal bovine serum (FBS, Gibco, Grand Island, NY, USA) at 37°C with 5% CO_2_ in 25-cm^2^ cell culture plates (Corning Inc., Corning, NY, USA). The bMECs were grown in the incubator for 24 h and passaged after reaching 60-70% fusion. At 24 h before the experiment, bMECs were seeded (1×10^5^ cells/mL) in 6-well plates (2 mL per well) or 96-well plates (0.1 mL per well) for *M. bovis* infection.

### Cell infection and gentamicin protection assay

The bMECs were grown in 6-well culture plates to reach 60-70% fusion, then infected (defined as 0 h) with *M. bovis* (multiplicity of infection [MOI]=1:30). After culture at 37°C for 1 h, cells were washed 3 times with sterile PBS (2 mL/well) to remove nonadherent *M. bovis*. Then, 2 mL of DMEM containing 400 μg/mL gentamicin (Solarbio, Beijing, China) was added to each well and incubated at 37°C for 2 h to kill extracellular *M. bovis* (Liu et al., 2021). Cells were again washed 3 times with sterile PBS (2 mL/well) and 2 mL of DMEM with 10% FBS and 10 μg/mL gentamicin was added to each well before experiments.

### Colony-forming unit assay

Using the method described above with *M. bovis*-infected cells, at indicated time points, cells were rinsed 3 times with PBS, detached with sterile scrapers, and resuspended in 500 μL of sterile PBS. The suspension was mixed by passing it through a syringe and 23G needle, followed by 10-fold serial dilutions. Dilutions were plated on *M. bovis* PPLOA medium and incubated at 37°C with 5% CO_2_ for up to 7 d. Each treatment had 3 replicate wells, with each well counted 3 times. Experiments were independently repeated 3 times.

### Transfection

Adenoviruses encoding GFP-LC3B and mCherry-GFP-LC3B fusion proteins (Ad-GFP-LC3B and Ad-mCherry-GFP-LC3B) are widely used for autophagy detection. These adenoviruses effectively label autophagosomes by expressing fusion proteins, facilitating real-time monitoring of autophagy. The bMECs were inoculated into 6-well culture plates as described above and cultured for 12 h to reach 40-50% fusion, when cells were infected using Ad-GFP-LC3B and Ad-mCherry-GFP-LC3B adenovirus mixed with DMEM containing 10% FBS at MOI = 1:4. Cells were grown in an incubator at 37°C and 5% CO_2_ for 24 h prior to subsequent experiments. GFP-LC3B labels autophagosomes whereas mCherry-GFP-LC3B detects autophagic flux.

### Protein extraction and western blotting

Nuclei and cytoplasmic proteins were extracted using nuclei and cytoplasmic protein extraction kits (Beyotime, Shanghai, China). To extract total cellular proteins, cells were washed 3 times with PBS at the indicated time points, and total proteins were extracted using RIPA lysis buffer (Beyotime) under ice-bath conditions. Collected proteins were centrifuged (12,000 × g for 20 min at 4°C), protein concentration was estimated using a BCA kit (Sigma Aldrich, B9643-1L) and samples were subjected to SDS-PAGE and transferred to PVDF (PolyVinyliDene Fluoride). Membranes were incubated with specific primary antibodies overnight at 4°C, then a peroxidase-coupled secondary antibody added, with incubation at room temperature for 1 h. Immunoreactivity was detected using the ECL detection system, and band densities analyzed with Image J (National Institutes of Health, Bethesda, MD, USA).

### RNA extraction, cDNA synthesis and real-time PCR

Cells were lysed with 1 mL of TransZol Up lysate per well, according to manufacturer’s instructions, and total RNA extracted according to the instructions of the Total RNA Extraction Kit (TransGen Biotech, Beijing, China). First-strand cDNA was synthesized by reverse transcription of RNA using One-Step gDNA Removal and cDNA Synthesis SuperMix (TransGen Biotech). RNA was reverse-transcribed using One-Step gDNA Removal and cDNA Synthesis SuperMix (TransGen Biotech). Real-time PCR was done on an Applied Biosystems StepOnePlus real time PCR (Thermo Fisher Scientific) system using SYBR Green PCR Master Mix (TransGen Biotech). Primer sequences were: Acetyl-CoA acetyltransferase 1 (*ACAT1*) (forward primer: AGGCGGGTGCAGGAAATAAG; reverse primer: ACCAAGTTTAGTGGCTGGCA), 3-hydroxy-3-methylglutaryl-CoA reductase (*HMGCR*) (forward primer: GCAGAGCAATAGGCCTTGGT; reverse primer: GAGTCACAAGCACGGGGAAA) and Sterol regulatory element binding transcription factor 2 (*SREBF2*) (forward primer: ATCGCTCCTCCATCAACGAC; reverse primer: CCTCAGAACGCCAGACTTGT) in real-time PCR.

### Cholesterol quantification

Infected cells were washed 3 times with PBS at designated time points, followed by addition of Buffer A for Metabolic Assay from the Amplex Red kit (Beyotime, Beijing, S0211S). Cells were gently resuspended to detach them and free cholesterol and cholesterol esters measured. Cholesterol standards were prepared according to manufacturer’s instructions, with a concentration range of 0 to 4 μg/mL (0 to 10 μM). An aliquot (50 μL) of cell lysate diluted with reaction buffer were added to the microplate, followed by addition of 50 μL of 300 μM Amplex Red working solution. The microplate was incubated at 3°C in the dark for 30 min. Fluorescence was measured using a fluorescence microplate reader (Thermo Fisher Scientific, USA) with an excitation wavelength of 560 nm and an emission wavelength of 590 nm.

### Confocal laser microscopy

*M. bovis* was stained with DiI (Beyotime, C1036), a red fluorescent cell membrane dye. The *M. bovis* pellet was resuspended in DMEM medium containing 5 μM DiI, vortexed to ensure uniform mixing, and incubated at room temperature for 20 min. The bacterial pellet was harvested by centrifugation at 8000 × g for 30 min, followed by washing twice with PBS for subsequent infection of bMECs. After treatment at designated time points, cells were washed 3 times with PBS, fixed with 4% paraformaldehyde (PFA, Solarbio) for > 4 h, permeabilized with 0.5% Triton X-100 (Sigma Aldrich, X100) for 10 min, and incubated 1 h at room temperature in PBS with 3% bovine serum albumin. Subsequently, cells were incubated overnight at 4°C with primary antibodies targeting TFEB and TFE3 (1:500). Following antibody incubation, cells were washed with sterile PBS and then incubated at room temperature with Cy3-conjugated goat anti-rabbit IgG (H+L) (Beyotime, A0516, 1:500) and Cy3-conjugated goat anti-mouse IgG (H+L) (Beyotime, A0521, 1:500) for 1 h each. Finally, nuclei were stained with Hoechst 33258 (Beyotime, C1017). Cell cholesterol was stained using Filipin complex; cell slides were immersed in a solution of 500 μg/mL Filipin complex in PBS at room temperature for 20 min. Coverslips were prepared using an anti-fluorescence quenching mounting medium (Beyotime, P0126), and observed under a Nikon A1 LFOV laser scanning confocal microscope at wavelengths of 405, 488 and 561 nm. mCherry and GFP puncta were quantified from 20 cells per sample and a minimum of 60 cells per group for statistical analysis. All experiments were performed independently at least 3 times (n ≥ 3), and data are presented as mean ± SD.

### Murine mammary infection model for *M. bovis*

To establish a pathogenic model of *M. bovis*-induced mastitis, specific pathogen-free (SPF) female Balb/c mice, 6–8 wk old (SiPeiFu Laboratory Animal Technology, Beijing, China) were used. Pregnant mice at gestational day 20 were housed in sterile isolators under controlled environmental conditions, with *ad libitum* access to feed and water and a 12-h light/dark cycle. On postpartum day 3, mice were anesthetized via intramuscular injection of 50 mg/kg Zoletil 50 (Virbac, Carros, France). The fourth pair of mammary glands was surgically exposed by excising the nipple tip, and 100 μL of *M. bovis* suspension (10^6 CFU) was slowly injected through a blunt needle (30 G, 25 mm) into the mammary duct. Mice were randomly assigned to 6 groups (n = 6 per group): 2 infection groups (inoculated with the PG45 reference strain or WT21 wild-type strain); 3 treatment groups (fenofibrate alone, PG45 + fenofibrate, WT21 + fenofibrate); and 1 negative control group (sterile PBS). Nursing pups were removed 2 h before intramammary inoculation. At 12 h post-infection, mice were weighed and treated with fenofibrate (100 mg/kg) via intraperitoneal injection. Fenofibrate was first dissolved in DMSO to prepare a stock solution, then diluted with 10% stock solution and 90% SBE-β-CD in saline, followed by ultrasonic heating to ensure complete solubilization. After 36 h of treatment, mice were anesthetized via intramuscular injection of Zoletil 50 (50 mg/kg) and subsequently euthanized by CO_2_ inhalation (displacement rate of 20-30% chamber volume per minute) followed by cervical dislocation to ensure death, and mammary tissues harvested to determine bacterial burden. Under sterile conditions, 0.1 g of mammary tissue was excised, homogenized in sterile tubes, and centrifuged (3000 g x 15 min). An aliquot (10 μL) of supernatant was serially diluted and plated onto PPLO solid medium. After incubation, viable colonies were counted and expressed as colony-forming units (CFU) per gram of tissue.

### Hematoxylin and eosin staining

Mice were anesthetized and euthanized 48 h post-inoculation with *M. bovis* or treatment. The fourth pair of mammary glands were aseptically excised, immersed in 75% alcohol for 3 min, and fixed in 4% paraformaldehyde. Tissues were dehydrated, embedded in paraffin, sectioned (5 μm), dewaxed in xylene, rehydrated through an alcohol series, stained with hematoxylin and eosin, and viewed under an optical microscope.

### Immunohistochemistry staining

Serial sections of paraffin-embedded mouse mammary tissues were deparaffinized with xylene and rehydrated through a graded ethanol series. Antigen retrieval involved boiling in citrate buffer (pH 6.0) for 15 min, then natural cooling to room temperature. Endogenous peroxidase activity was blocked by incubating in 3% H_2_O_2_ in methanol at room temperature for 10 min. After 3 washes, sections were blocked with 5% goat serum at 37 °C for 30 min. Primary antibodies against LC3B, LAMP1, LAMP2, and SQSTM1 were applied and incubated overnight at 4 °C in a humidified chamber. After 3 washes with PBS buffer, secondary antibodies were added and incubated. Immunoreactivity was visualized using 3,3’-diaminobenzidine (DAB) substrate solution, with development time microscopically monitored. Sections were counterstained with hematoxylin, dehydrated, and cleared. Finally, slides were mounted with neutral resin, and immunohistochemical (IHC) micrographs were captured using a light microscope.

### Detection of cytokines in murine mammary gland

Mammary tissues were accurately weighed, placed into 1.5 mL centrifuge tubes containing precooled PBS (pH 7.4), stainless steel beads were added and samples homogenized using a high-throughput tissue grinder (60 Hz, 2 min, twice). Homogenates were centrifuged at 5000 × g for 10 min at 4 °C, and supernatants collected. Concentrations of tumor necrosis factor (TNF-α), interleukin 1β (IL-1β), interleukin 6 (IL-6), and interleukin 10 (IL-10) in supernatants were determined using ELISA kits (Enzyme-linked Biotechnology Co., Ltd., Shanghai, China), in accordance with manufacturer’s instructions.

### Statistical analyses

Data are mean ± standard deviation (SD) from ≥ 3 independent experiments. Statistical analyses were performed using SPSS 26.0 (IBM Corp., Armonk, NY, USA), with GraphPad Prism 8.0 (GraphPad Software, San Diego, CA, USA) used for graphical representations. For parametric data, 1- or 2-way analysis of variance (ANOVA) was applied, followed by Tukey’s (1-way) or Bonferroni’s (2-way) *post hoc* tests. Significance thresholds were: *P < 0.05, **P < 0.01, and ***P < 0.001.

## Results

### Fenofibrate modulates autophagy in bMECs in concentration- and time-dependent manners

**Figure 1:**
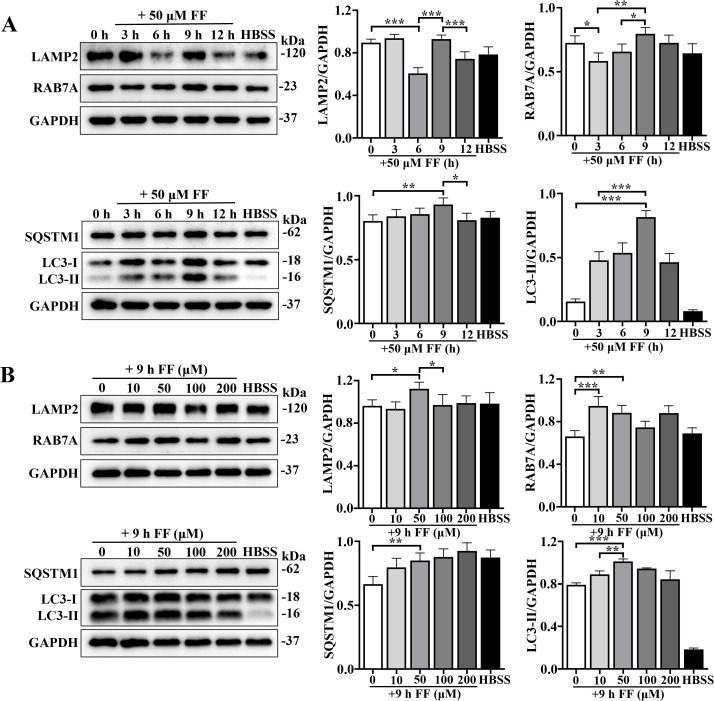
Effects of doses and treatment durations of fenofibrate on autophagy by bMECs. **(A)** bMECs were treated with 50 μM fenofibrate for 0, 3, 6, 9, or 12 h, with HBSS serving as a positive control. Expression levels of lysosome-associated proteins LAMP2 and RAB7A, along with autophagy markers LC3 and SQSTM1, were assessed by Western blotting. Densitometric analysis was performed to quantify protein expression, normalized to GAPDH as a loading control. **(B)** bMECs were treated with 0, 10, 50, 100, or 200 μM fenofibrate for 9 h, with HBSS as a positive control. Protein levels of LAMP2, RAB7A, LC3, and SQSTM1 were evaluated by Western blotting. Relative expression was quantified by densitometry and normalized to GAPDH (for **(A, B)**, 1-way ANOVA Dunnett’s multiple comparisons tests and 2-tailed unpaired *t*-tests were used). Data are presented as mean ± SD from 3 independent experiments. *P < 0.05; **P < 0.01; ***P < 0.001.

Western blotting was used to optimize concentration and treatment duration of fenofibrate for autophagy induction in bovine mammary epithelial cells (bMECs), using HBSS treatment as a positive control. Treatment with 50 μM fenofibrate for 9 h significantly upregulated expression of lysosomal membrane-associated protein LAMP2 and lysosomal transport and maturation protein RAB7A, compared to the previous time point. Concurrently, autophagosome-associated marker LC3B (*P* < 0.001) and autophagy receptor protein SQSTM1 (*P* = 0.008) were also increased in a time-dependent manner (**Figure 1A**). Furthermore, treatment with 50 μM fenofibrate for 9 h resulted in the most significant enhancement of LAMP2 (*P* = 0.028), RAB7A (*P* = 0.001), LC3B (*P* < 0.001), and SQSTM1 (*P* = 0.004) expression (**Figure 1B**). Therefore, 50 μM for 9 h was used for fenofibrate-induced autophagy in bMECs.

### Fenofibrate enhances autophagic activity in *M. bovis*-infected bMECs and restores autophagic flux

**Figure 2:**
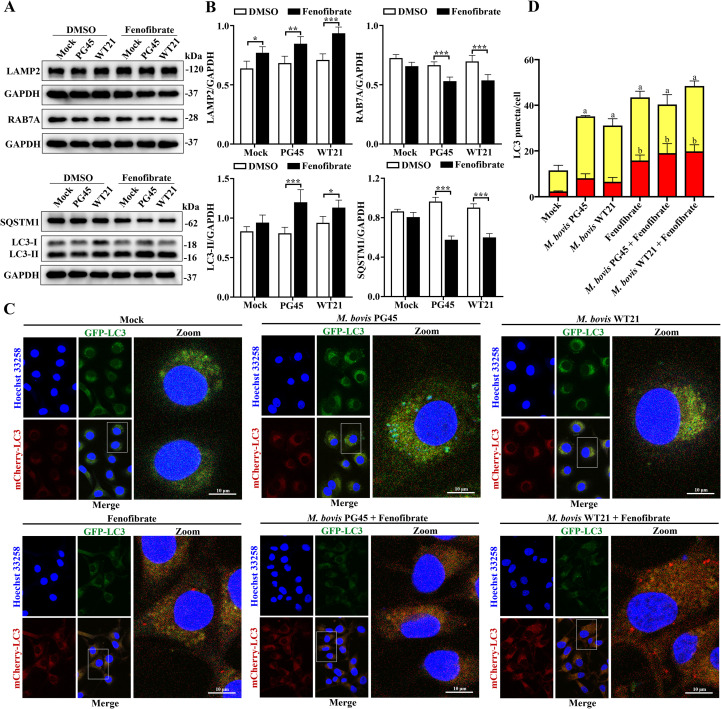
Fenofibrate restores autophagic activity and autophagic flux in bMECs infected with *M. bovis*. **(A)** bMECs were divided into 6 experimental groups: control, *M. bovis* PG45 reference strain infection (MOI = 30, 9 hpi), *M. bovis* WT21 wild-type strain infection (MOI = 30, 9 hpi), fenofibrate treatment alone, fenofibrate combined with PG45 infection, and fenofibrate combined with WT21 infection. Expression levels of LAMP2, RAB7A, SQSTM1, and LC3, along with the loading control GAPDH, were evaluated by Western blot (using specific antibodies). **(B)** Densitometric analysis was performed to quantify relative protein levels of LAMP2, RAB7A, SQSTM1, and LC3-II, normalized to GAPDH. **(C)** Representative confocal images depict autophagic flux in mCherry-GFP-LC3-transfected bMECs across the 6 treatment groups: control, PG45-infected, WT21-infected, fenofibrate-treated, fenofibrate+PG45, and fenofibrate+WT21. Yellow puncta indicate autophagosomes, whereas red puncta represent autolysosomes. Nuclei were counterstained with Hoechst 33258 (blue). Scale bar = 10 μm. **(D)** Quantification of autophagosomes in bMECs. Twenty cells for each sample and at least 60 cells in each group were used for statistical analyses, Superscript ‘a’: Yellow puncta compared to the control group, Superscript ‘b’: Red puncta compared to the control group. For **(A, B)**, 2-way ANOVA Dunnett’s multiple comparisons tests were used; for **(D)**, 1-way ANOVA Dunnett’s multiple comparisons tests and 2-tailed unpaired *t*-tests were used. Data are presented as mean ± SD from 3 independent experiments. *P < 0.05; **P < 0.01; ***P < 0.001.

To investigate whether fenofibrate modulates autophagy in bMECs through regulation of autophagy and lysosomal activity during *M. bovis* infection, *in vitro* infection models were established using *M. bovis* standard strain PG45 and wild-type strain WT21. In the *M. bovis*-infected group, Western blotting results indicated that fenofibrate markedly upregulated expression of lysosomal membrane protein LAMP2 and LC3B (autophagy marker), but downregulated lysosomal trafficking protein RAB7A (*P* < 0.001) and autophagy receptor SQSTM1 (*P* < 0.001). As SQSTM1 binds to autophagic substrates and is degraded in lysosomes, its reduction is a key indicator of autophagic flux integrity (**Figures 2A, B**). To further assess effects of fenofibrate on autophagic flux, confocal microscopy was used to examine cells transfected with mCherry-GFP-LC3B. Upon autophagosome-lysosome fusion, the lysosomal environment quenches the GFP signal, leaving red fluorescence. Quantification of red and yellow puncta yielded no significant increase in red puncta in the *M. bovis*-infected group compared to controls, indicating impaired lysosomal cargo transport during infection. Fenofibrate treatment increased both red puncta (*P* < 0.001) and yellow puncta (*P* < 0.001) relative to controls, even with *M. bovis* infections (2 strains), indicating enhanced autophagic flux (**Figures 2C, D**).

### Fenofibrate promotes nuclear translocation of TFEB and TFE3 to modulate autophagic responses in bMECs during *M. bovis* infection

**Figure 3:**
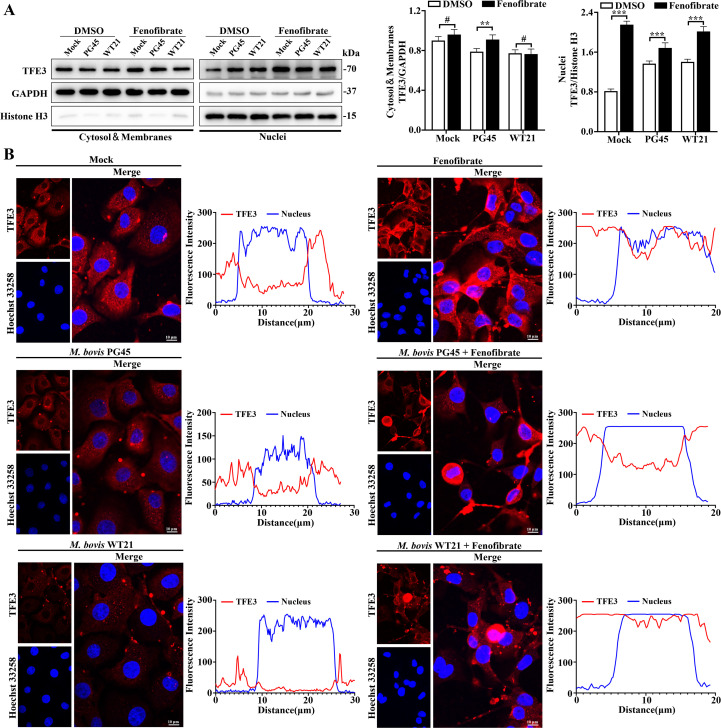
Fenofibrate induces nuclear translocation of TFE3 in bMECs infected with *M. bovis*. **(A)** bMECs were divided into 6 groups: control, *M. bovis* PG45 strain infection, *M. bovis* WT21 wild-type strain infection, fenofibrate-treated, fenofibrate + PG45 infection, and fenofibrate + WT21 infection. Western blot analysis was used to evaluate TFE3 expression in cytoplasmic and nuclear fractions. Relative protein levels were quantified by densitometry and normalized to GAPDH (cytoplasm) or Histone H3 (nucleus). **(B)** Immunofluorescence staining was conducted to examine subcellular localization of endogenous TFE3 (red) in bMECs across the 6 groups. Nuclei were stained with Hoechst 33258 (blue). Representative confocal images are shown. Scale bar = 10 μm. For **(A)** 2-way ANOVA Dunnett’s multiple comparisons tests were used. Data are presented as mean ± SD from 3 independent experiments. #*P* > 0.05, *P < 0.05; **P < 0.01; ***P < 0.001.

**Figure 4:**
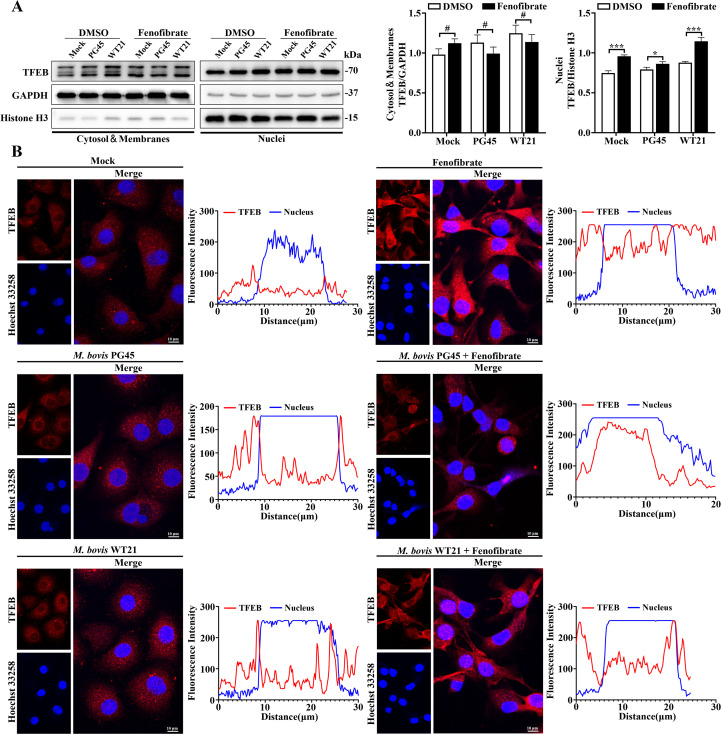
Fenofibrate induces nuclear translocation of TFEB in bMECs infected with *M. bovis*. **(A)** bMECs were divided into 6 groups: control, *M. bovis* PG45 strain infection, *M. bovis* WT21 wild-type strain infection, fenofibrate-treated, fenofibrate + PG45 infection, and fenofibrate + WT21 infection. Western blot analysis was used to evaluate TFEB expression in cytoplasmic and nuclear fractions. Relative protein levels were quantified by densitometry and normalized to GAPDH (cytoplasm) or Histone H3 (nucleus). **(B)** Immunofluorescence staining was conducted to examine subcellular localization of endogenous TFEB (red) in bMECs across the 6 groups. Nuclei were stained with Hoechst 33258 (blue). Representative confocal images are shown. Scale bar = 10 μm. For **(A)**, 2-way ANOVA Dunnett’s multiple comparisons tests were used. Data are presented as mean ± SD from 3 independent experiments. #*P* > 0.05, *P < 0.05; **P < 0.01; ***P < 0.001.

To investigate how fenofibrate regulates autophagy in bMECs, we assessed expression and nuclear translocation TFEB and TFE3, key transcriptional regulators of lysosome biogenesis and autophagy. Fenofibrate increased cytoplasmic TFE3 expression in the *M. bovis* PG45-infected group (*P* = 0.005; **Figure 3A**), with minimal impact on cytoplasmic TFEB (**Figure 4A**). Furthermore, fenofibrate promoted nuclear accumulation of TFEB and TFE3 (*P* ≤ 0.011; **Figures 4A**, **3A**). Confocal imaging corroborated these findings, with increased TFEB and TFE3 fluorescence intensity following fenofibrate exposure (**Figures 4B**, **3B**). Notably, under *M. bovis* infection, both TFEB and TFE3 were predominantly localized in the cytoplasm, whereas fenofibrate enhanced their translocation into the nucleus (**Figures 4B**, **3B**). Therefore, fenofibrate promoted TFEB and TFE3 nuclear localization, upregulating transcription of autophagy- and lysosome-related genes and enhancing autophagic activity in bMECs.

### Fenofibrate modulates cholesterol production during *M. bovis* infection

**Figure 5:**
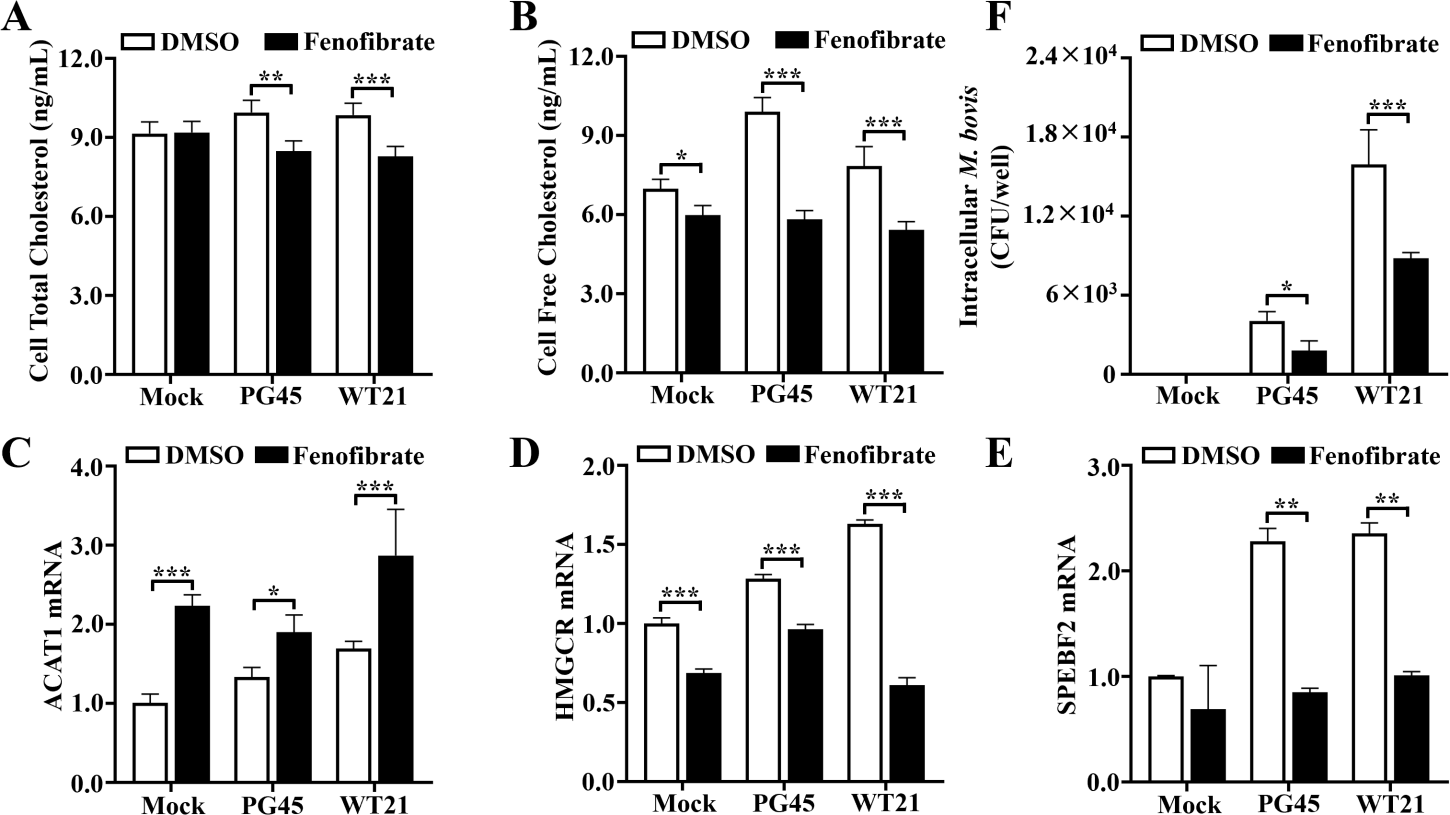
Fenofibrate affects free and total cholesterol concentrations, cholesterol-related gene transcription, and intracellular *M. bovis* load. **(A)** Total cholesterol concentrations in bMECs were quantified using the Amplex™ Cholesterol Assay Kit across 6 groups: control, *M. bovis* PG45 strain infection, *M. bovis* WT21 wild-type strain infection, fenofibrate treatment, fenofibrate + PG45 infection, and fenofibrate + WT21 infection. **(B)** Free cholesterol content in bMECs from 6 groups was measured using the Amplex™ Cholesterol Assay Kit. **(C–E)** The mRNA expression levels of cholesterol esterification gene ACAT1 and key cholesterol biosynthesis regulators HMGCR and SREBF2 were determined by quantitative real-time PCR and normalized to β-actin. **(F)** Bacterial burden was assessed by plating cell lysates from each group onto PPLOA agar, and colony-forming units (CFU) enumerated. For **(A–F)**, 2-way ANOVA Dunnett’s multiple comparisons tests were used. Data are presented as mean ± SD from 3 independent experiments. *P < 0.05; **P < 0.01; ***P < 0.001.

We evaluated effects of fenofibrate on intracellular free and total cholesterol, as well as expression of cholesterol-regulating genes (ACAT1, HMGCR, SREBF2) in *M. bovis*-infected bMECs. Infection elevated intracellular free cholesterol concentrations (*P* ≤ 0.044), whereas fenofibrate reduced both total and free cholesterol compared to infected controls (*P* ≤ 0.001; **Figures 5A, B**). Notably, fenofibrate upregulated transcription of ACAT1, a gene involved in esterification of free cholesterol with acyl-CoA, whereas expression of HMGCR, the rate-limiting enzyme in cholesterol biosynthesis, and its upstream transcriptional activator SREBF2 were downregulated (*P* ≤ 0.024; **Figures 5C–E**). Concurrently, fenofibrate decreased intracellular colony-forming units (CFUs) of both PG45 and WT21 strains (*P* ≤ 0.012; **Figure 5F**). Therefore, fenofibrate modulated both free and total cholesterol concentrations and regulated transcription of cholesterol-associated genes during *M. bovis* infection, thereby attenuating intracellular bacterial burden.

### Fenofibrate modulates colocalization of cholesterol and autophagy marker LC3 during *M. bovis* infection

**Figure 6:**
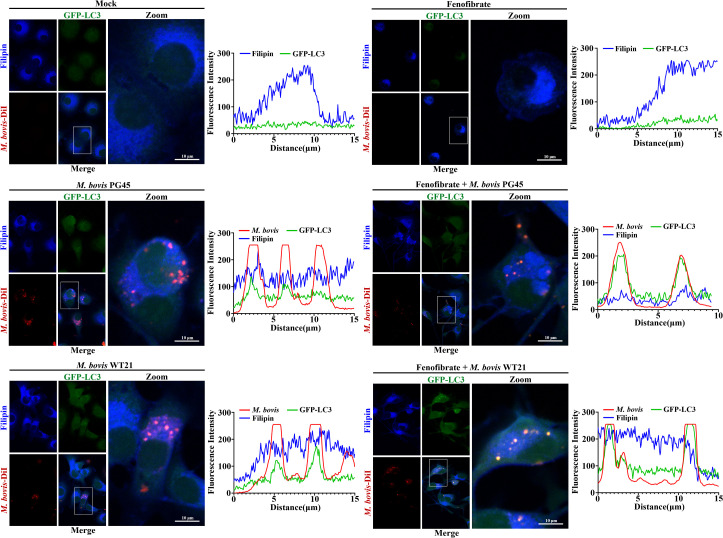
Fenofibrate affects co-localization of *M. bovis*, cholesterol, and LC3. **(A)** bMECs were divided into 6 groups: control, *M. bovis* PG45-infected, *M. bovis* WT21 wild-type strain-infected, fenofibrate-treated, fenofibrate + PG45-infected, and fenofibrate + WT21-infected. Representative confocal images illustrate triple staining with DiI to label *M. bovis* (red), GFP-LC3 to visualize the autophagy marker LC3 (green), and Filipin to detect cholesterol (blue). Scale bar = 10 μm.

Alterations in intracellular cholesterol concentrations can inhibit maturation of autolysosomes, thereby modulating autophagic activity. In this study, intracellular free cholesterol was visualized using Filipin staining, *M. bovis* was labeled with red fluorescence, and the autophagy marker LC3 was monitored via GFP-LC3 adenoviral transfection. Colocalization among cholesterol, *M. bovis*, and LC3 was assessed using confocal laser microscopy. Infection with *M. bovis* PG45 or WT21 reduced colocalization between autophagosomes (green) and *M. bovis* (red), as indicated by a decreased proportion of yellow puncta. Concurrently, colocalization between *M. bovis* and free cholesterol increased, as evidenced by enhanced intensity of pink puncta. Fenofibrate diminished *M. bovis*-cholesterol colocalization (reduced pink puncta) and restored autophagosome-*M. bovis* colocalization (increased yellow puncta), thereby limiting bacterial access to host free cholesterol and suppressing *M. bovis* intracellular replication, as reflected by decreased red fluorescence intensity (**Figure 6**).

### Fenofibrate modulates expression of autophagy and lysosome-associated proteins in murine mammary tissue during *M. bovis* infection

**Figure 7:**
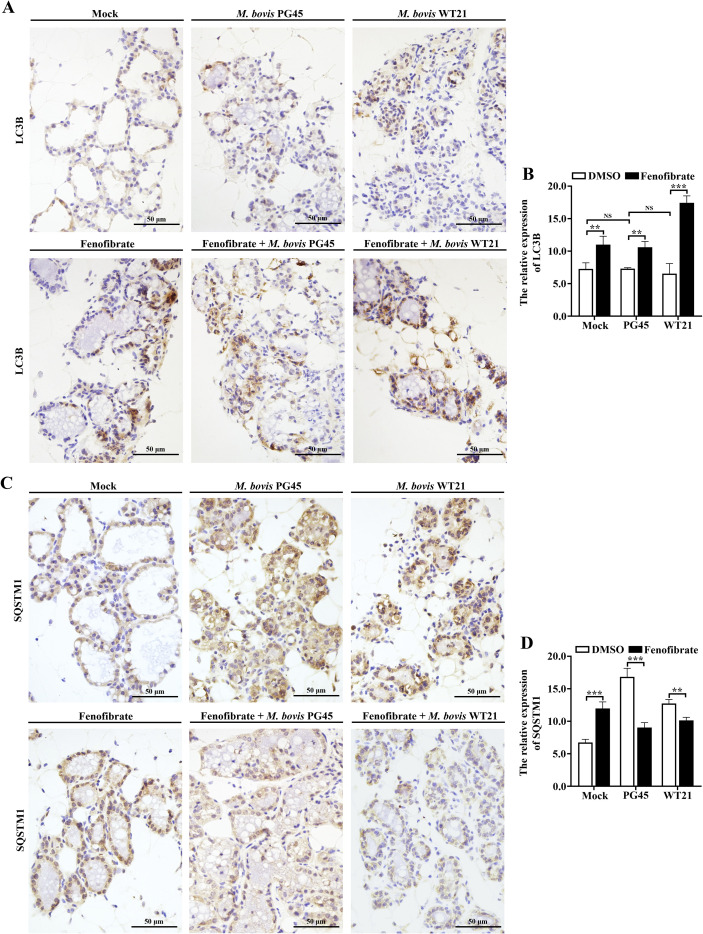
Fenofibrate affects expression of autophagy markers in mammary tissue of mice infected with *M. bovis*. **(A)** Mice were allocated into 6 groups: control, *M. bovis* PG45-infected, *M. bovis* WT21 wild-type strain-infected, fenofibrate-treated, fenofibrate + PG45-infected, and fenofibrate + WT21-infected. Immunohistochemical staining was used to detect microtubule-associated protein 1 light chain 3 beta (LC3B) expression in murine mammary tissue. LC3B-positive granule-like cells were observed under a light microscope, with brown staining in nuclei indicating positive signals. **(B)** Quantification of LC3B-positive staining intensity in mammary tissues. **(C)** Using the same 6 experimental groups, immunohistochemical staining was performed to assess SQSTM1 expression in mammary tissue. SQSTM1-positive granule-like cells were observed under a light microscope, with brown nuclear staining indicating positive expression. **(D)** Quantification of SQSTM1-positive staining intensity in mouse mammary tissues. Scale bar = 50 μm. For **(B, D)**, 2-way ANOVA Dunnett’s multiple comparisons tests were used. Data are presented as mean ± SD from 3 independent experiments. NS (Not Significant) *P* > 0.05; *P < 0.05; **P < 0.01; ***P < 0.001.

**Figure 8:**
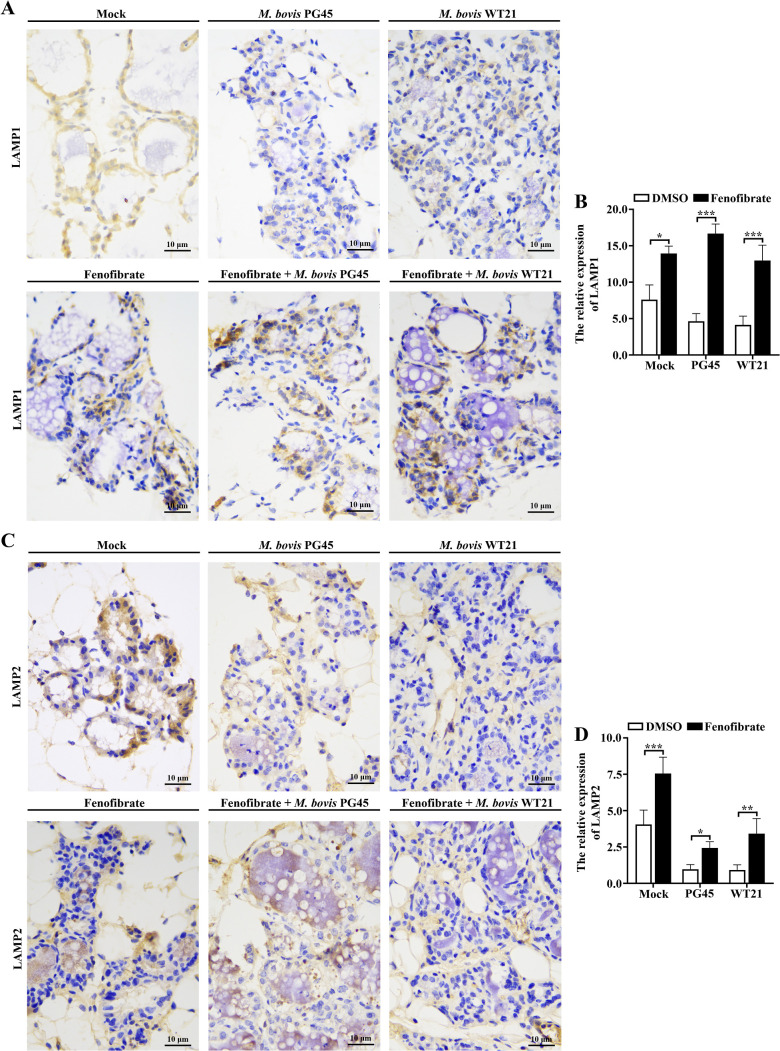
Fenofibrate affects expression of lysosome markers in mammary tissue of mice infected with *M. bovis*. **(A)** Mice were allocated into 6 groups: control, *M. bovis* PG45-infected, *M. bovis* WT21 wild-type strain-infected, fenofibrate-treated, fenofibrate + PG45-infected, and fenofibrate + WT21-infected. Immunohistochemical staining was used to detect LAMP1 expression in murine mammary tissue. LAMP1-positive granule-like cells were observed under a light microscope, with brown staining in nuclei indicating positive signals. **(B)** Quantification of LAMP1-positive staining intensity in mammary tissues. **(C)** Using the same 6 experimental groups, immunohistochemical staining was performed to assess LAMP2 expression in mammary tissue. LAMP2-positive granule-like cells were observed under a light microscope, with brown nuclear staining indicating positive expression. **(D)** Quantification of LAMP2-positive staining intensity in mammary tissues. Scale bar = 10 μm. For **(B, D)**, 2-way ANOVA Dunnett’s multiple comparisons tests were used. Data are presented as mean ± SD from 3 independent experiments. *P < 0.05; **P < 0.01; ***P < 0.001.

To assess *in vivo* therapeutic efficacy of fenofibrate against *M. bovis* infection, a Balb/C murine mastitis model was established. Immunohistochemistry of mammary tissues was done to determine whether fenofibrate mitigated *M. bovis*-induced mastitis by modulating autophagy and lysosomal activity. Compared to the control group, infection with *M. bovis* strains PG45 and WT21 did not significantly alter expression of the autophagy marker LC3B (*P* ≥ 0.405), indicating no evident autophagy. Notably, fenofibrate treatment restored LC3B (*P* ≤ 0.003) expression and reduced SQSTM1 in the *M. bovis*-infected group (*P* ≤ 0.002) accumulation, suggesting a reactivated autophagic flux (**Figures 7A–D**). Furthermore, expression of lysosomal membrane proteins LAMP1 and LAMP2 was decreased following *M. bovis* infection (*P* ≤ 0.035), whereas fenofibrate upregulated both proteins compared to the infected group (*P* ≤ 0.043; **Figures 8A–D**).

### Fenofibrate alleviates *M. bovis*-induced mammary tissue damage and intracellular bacterial load by modulating cholesterol homeostasis

**Figure 9:**
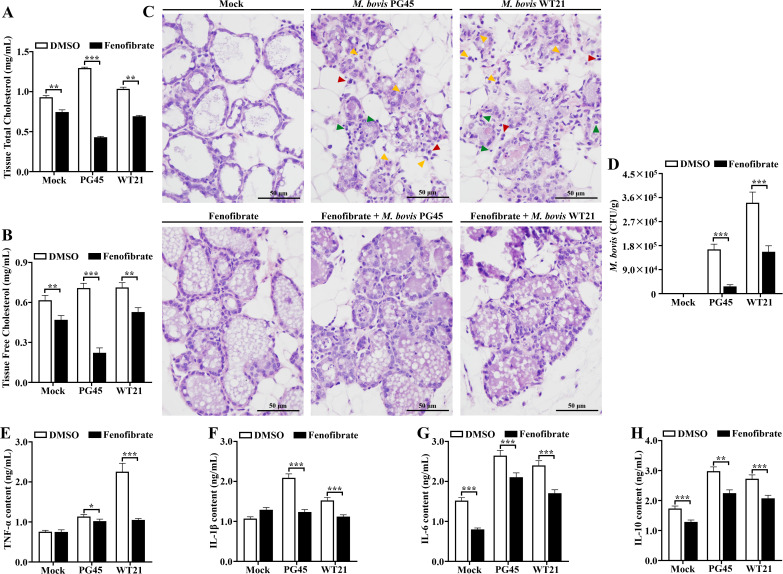
Fenofibrate affects cholesterol concentrations, *M. bovis* load, tissue morphology, and cytokine concentrations in murine mammary glands. **(A)** Total cholesterol concentrations in murine mammary glands were quantified using the Amplex™ Cholesterol Assay Kit across 6 experimental groups: control, *M. bovis* PG45-infected, *M. bovis* WT21 wild-type strain-infected, fenofibrate-treated, fenofibrate + PG45-infected, and fenofibrate + WT21-infected. **(B)** Free cholesterol content in mammary tissues from the same 6 groups was determined using the Amplex™ Cholesterol Assay Kit. **(C)** Hematoxylin and eosin (H&E) staining was performed on mammary gland sections from all 6 groups to assess tissue architecture and histopathological changes. Representative images under light microscopy highlight neutrophils (yellow arrows), lymphocytes (green arrows), and plasma cells (red arrows). **(D)** Bacterial load was evaluated by plating serial dilutions of tissue lysates from each group onto PPLOA agar, followed by enumeration of colony-forming units (CFU). Scale bar = 50 μm. **(E)** content of TNF-α in mammary gland tissue. **(F)** content of IL-1β in mammary gland tissue. **(G)** content of IL-6 in mammary gland tissue. **(H)** content of IL-10 in mammary gland tissue. For **(A, B, D-H)**, 2-way ANOVA Dunnett’s multiple comparisons tests were used. Data are presented as mean ± SD from 3 independent experiments. *P < 0.05; **P < 0.01; ***P < 0.001.

To assess impacts of fenofibrate treatment on cholesterol in mammary tissues during *M. bovis* infection, an Amplex™ Cholesterol Assay Kit was used. Total and free cholesterol concentration in mammary glands were significantly elevated at 48 h post-infection (hpi) compared to uninfected controls. Intraperitoneal administration of fenofibrate (100 mg/kg) for 36 h reduced both total and free cholesterol concentrations in infected tissues relative to the untreated infection group (*P* < 0.01; **Figures 9A, B**). Histopathological evaluation demonstrated pronounced inflammatory cell infiltration in the lumen of mammary alveoli at 48 hpi, with accumulation of neutrophils (yellow arrows), lymphocytes (green arrows), and plasma cells (red arrows), with the latter 2 indicative of a chronic inflammatory response. In addition, desquamated epithelial cells, presumably undergoing necrosis, were observed within the lumen of mammary alveoli. In contrast, fenofibrate-treated mice had intact mammary gland architecture and negligible inflammatory cell infiltration (**Figure 9C**).

To quantify bacterial burden, *M. bovis* was isolated from mammary tissues and colony-forming units (CFU) enumerated. At 48 hpi, PG45 and WT21 strains reached average burdens of 1.5 × 10^4 CFU/g and 2.4 × 10^4 CFU/g, respectively. Notably, fenofibrate reduced intramammary *M. bovis* load (*P* < 0.001; **Figure 9D**) and decreased mammary expression of pro-inflammatory cytokines TNF-α (*P* < 0.05; **Figure 9E**), IL-1β (*P* < 0.001; **Figure 9F**), and IL-6 (*P* < 0.001; **Figure 9G**), and the anti-inflammatory cytokine IL-10 (*P* < 0.001; **Figure 9H**), compared to PG45 and WT21-infected groups.

## Discussion

This study demonstrates that fenofibrate, a PPARα agonist, effectively combats *M. bovis* infection by restoring autophagic flux and reprogramming host cholesterol metabolism.

Increasing evidence highlights the pivotal role of host lipid metabolism and autophagic pathways in determining the fate of intracellular bacterial pathogens (Van Wyngene et al., 2020; Pays, 2024). In this study, fenofibrate, a peroxisome proliferator-activated receptor alpha (PPARα) agonist, effectively suppressed *M. bovis* replication and infection (both *in vitro* and *in vivo*) by restoring autophagic flux and reprogramming intracellular cholesterol homeostasis. Furthermore, fenofibrate enhanced autophagy in *M. bovis*-infected bovine mammary epithelial cells (bMECs) and murine mammary tissue, promoted nuclear translocation of lysosomal transcription factors TFEB and TFE3, and facilitated lysosomal biogenesis and functional maturation. These processes markedly reduced intracellular free and total cholesterol concentrations, with transcriptional modulation of cholesterol metabolism-related genes, restricting *M. bovis* replication within host cells. By assessing cholesterol metabolism, autophagy regulation, and bacterial intracellular persistence, this study offers novel insights into host-pathogen interactions and identifies fenofibrate as a promising therapeutic candidate for *M. bovis*-induced bovine mastitis via cholesterol-targeted intervention.

In previous studies, fenofibrate modulated autophagy and lysosomal function in host cells (Yoo et al., 2021). To optimize concentration and treatment duration for fenofibrate-induced autophagy in bMECs, Western blotting was used to evaluate autophagy-related protein expression. Fenofibrate induced autophagy in bMECs in a time- and dose-dependent manner, with upregulation of canonical autophagy markers LC3B and SQSTM1, along with lysosomal proteins LAMP2 and RAB7A, consistent with a report that fenofibrate enhanced autophagy (Cetti et al., 2025). The optimal treatment for inducing autophagy was 50 μM for 9 h. Increased expression of LAMP2 and RAB7A implied that fenofibrate not only promoted autophagosome formation but also facilitated lysosomal maturation, a critical step in activation of autophagic flux (Kimmey and Stallings, 2016). Furthermore, *M. bovis* infection failed to enhance autophagic flux in bMECs, with accumulation of the autophagy receptor SQSTM1, a well-established marker of impaired autophagic degradation (Lamark et al., 2017). Similarly, certain bacterial pathogens (including *Listeria monocytogenes*, *Salmonella enterica*, *Mycobacterium tuberculosis*, and *M. bovis*), can subvert host autophagy to evade immune clearance (Kang et al., 2022). Notably, treatment with fenofibrate markedly reversed these alterations, leading to increased expression of the lysosomal membrane protein LAMP2 and the autophagy marker LC3B, accompanied by a pronounced reduction in SQSTM1. However, downregulation of RAB7A expression may reflect a negative feedback mechanism activated upon restoration-or even enhancement-of autophagic flux, serving to prevent excessive autophagy and potential cellular damage. Furthermore, confocal microscopy of mCherry-GFP-LC3-transfected cells confirmed that fenofibrate enhanced autophagosome-lysosome fusion and activated autophagic flux, even in the context of *M. bovis* infection. Therefore, fenofibrate restored impaired autophagy by relieving *M. bovis*-induced blockade of autophagic flux.

The MiT/TFE family of transcription factors is a crucial group of signaling molecules that regulate autophagy and lysosomal function. Among these, TFEB and TFE3 are involved in modulation of various cellular processes. We reported that promoting nuclear translocation of TFEB in bMECs effectively restored impaired autophagic responses during *M. bovis* infection (Xu et al., 2024). In the present study, to further elucidate regulatory mechanisms by which fenofibrate modulates autophagy, we examined expression and subcellular localization of TFEB and TFE3, 2 transcription factors pivotal to the autophagy-lysosome axis. Fenofibrate markedly promoted nuclear translocation of both TFEB and TFE3, whereas *M. bovis* infection led to their cytoplasmic sequestration. Therefore, we inferred that fenofibrate acts upstream of autophagy-related gene expression by modulating TFEB and TFE3 activity. Involvement of TFEB and TFE3 aligned with reports that fibrates can induce their nuclear localization, thereby activating lipophagic programs (Jarocki et al., 2024). Thus, fenofibrate may restore host antimicrobial capacity and promote pathogen clearance by transcriptionally reprogramming the autophagy-lysosome network.

Cholesterol is increasingly recognized as a pivotal determinant of intracellular bacterial growth and virulence. Several pathogens hijack host lipid metabolism, particularly by disrupting phosphoinositide signaling pathways and exploiting host-derived lipids, especially cholesterol, as essential nutrients (Walpole et al., 2018). *Mycoplasma* spp., owing to their highly reduced genomes, are particularly dependent on exogenous cholesterol, derived from the host or culture media, for replication and survival (Adamu et al., 2020). In the present study, *M. bovis* infection markedly elevated intracellular free cholesterol concentration in bMECs, potentially facilitating membrane integrity and intracellular replication of *M. bovis*. In contrast, fenofibrate significantly reduced both total and free cholesterol concentrations while concurrently altering the transcriptional profile of cholesterol metabolism-related genes. Specifically, fenofibrate upregulated ACAT1, which catalyzes cholesterol esterification, and downregulated key regulators of cholesterol biosynthesis, HMGCR and SREBF2. These transcriptional shifts were accompanied by a substantial decrease in intracellular *M. bovis* burden, suggesting that fenofibrate can interfere with cholesterol metabolism and affect intracellular survival of *M. bovis*. Furthermore, confocal microscopy using triple labeling for cholesterol, LC3, and *M. bovis* revealed reduced co-localization between the pathogen and free cholesterol following fenofibrate treatment, alongside a concomitant increase in its association with autophagosomes. Therefore, fenofibrate-mediated redistribution of cholesterol may promote autophagic clearance of *M. bovis*. Other intracellular pathogens exhibit similar cholesterol dependence. For example, inhibition of cholesterol retrograde transport impairs growth of *Chlamydia trachomatis*, likely due to failed cholesterol delivery to its inclusion bodies (Zhong et al., 2022). *Salmonella enterica* replicates within host cells inside Salmonella-containing vacuoles (SCVs), relying on 2 type III secretion system (TTSS) effectors, SseJ and SseL, to reroute cholesterol to SCVs due to inability to synthesize it (Raj et al., 2025). *Mycobacterium tuberculosis* uses host cholesterol as a carbon source; its acquisition and catabolism are essential for growth and persistence during chronic infection (Lovewell et al., 2016). Collectively, these findings underscored the therapeutic potential of targeting host cholesterol metabolism to control intracellular bacterial infections.

The protective efficacy of fenofibrate was further substantiated in a murine model of mastitis induced by *M. bovis* infection. This study employed a 48 h murine intramammary infection model to evaluate the efficacy of fenofibrate. The model was chosen because *M. bovis* induces acute inflammatory responses within 24–48 h, allowing intervention to capture early, pronounced pathological changes (Schneider et al., 2022). Moreover, fenofibrate primarily acts by modulating host cholesterol metabolism and autophagy, with its effects most critical during the early stages of infection (Yoo et al., 2021). Our results demonstrated that fenofibrate markedly reduced inflammatory cell infiltration in mammary tissues and preserved structural integrity of mammary alveoli. In parallel, fenofibrate significantly suppressed mammary expression of pro-inflammatory cytokines TNF-α, IL-1β, and IL-6, as well as the anti-inflammatory cytokine IL-10, indicating an overall dampening of tissue inflammation. Consistent with *in vitro* findings, fenofibrate restored expression of autophagy- and lysosome-associated markers LC3B and LAMP1/2 in infected mammary tissue and promoted degradation of SQSTM1, suggesting reactivation of autophagic and lysosomal function *in vivo*. However, significantly elevated SQSTM1 concentrations in the fenofibrate-treated group compared to controls may reflect delayed clearance of autophagic substrates in uninfected mammary tissue. Furthermore, the possibility of SQSTM1 upregulation via non-autophagic mechanisms, such as activation of the Nrf2 pathway (Zou et al., 2025), cannot be excluded and requires further study. Moreover, fenofibrate significantly decreased cholesterol accumulation within mammary tissue and reduced *M. bovis* colony-forming units (CFU), highlighting its ability to inhibit intracellular replication of *M. bovis* by regulating host cholesterol metabolism and autophagy.

Metabolic regulatory effects of fenofibrate can be mediated not only through its classical target PPARα but also by activating the AMPK pathway, which regulates protein synthesis and autophagy via mTORC1; this multi-target mechanism reduces the risk of rapid resistance development (Cheng et al., 2019). The involvement of additional pathways, such as AMPK, indicates potential off-target effects that may affect cellular processes beyond cholesterol metabolism. Notably, fenofibrate, akin to dexamethasone, has been demonstrated in both murine and human studies to suppress airway inflammation and proinflammatory cytokine release, including TNF-α, IL-1, and IFN-γ (Paw et al., 2018; Jin et al., 2023). Given its established clinical safety, fenofibrate may undergo more complex clinical evaluation for veterinary use. Although this study strongly indicates that fenofibrate exerts protective effects against *M. bovis* infection through autophagy-mediated cholesterol regulation, several limitations remain. Firstly, it is necessary to further validate its mechanism of action through PPARα knockout or interference experiments. Secondly, potential impacts of long-term fenofibrate use on physiological functions of mammary tissue, particularly lactation, requires thorough evaluation. Finally, further validation in bovine primary immune cell systems and *in vivo* models is still mandatory. Additionally, this study only used 2 strains, which may not fully reflect responses among various strains. Future studies should include more clinical isolates to verify the broad applicability of fenofibrate’s therapeutic effect, and systematically evaluate dose- and duration-dependent effects on milk production to ensure its safe veterinary application.

## Conclusions

**Figure 10:**
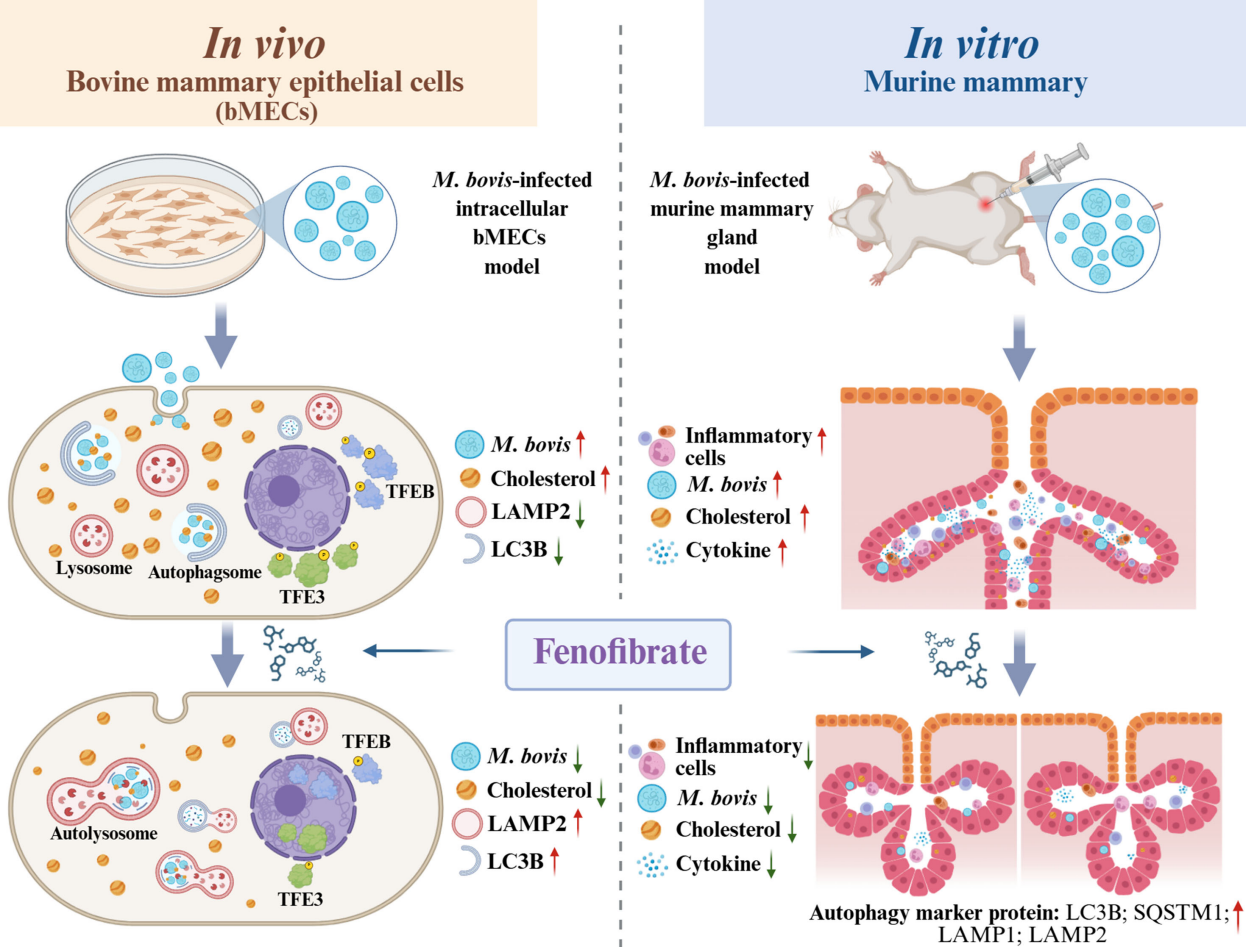
Fenofibrate suppresses *M. bovis* infection via autophagy-mediated cholesterol regulation in bovine and murine mammary tissue. Fenofibrate suppresses *M. bovis* infection by activating the TFE3 pathway to enhance autophagy, which in turn reduces bacterial load and normalizes cholesterol levels, ultimately alleviating disease in both cellular and animal models. This graphical abstract was created using BioRender (https://biorender.com).

Our findings revealed that fenofibrate effectively inhibited *M. bovis* infection by restoring autophagy and regulating intracellular cholesterol homeostasis. In bMECs, fenofibrate enhanced autophagy and lysosomal activity, promoted nuclear translocation of TFEB and TFE3, and rescued autophagic flux impaired by *M. bovis*. It also reduces cholesterol accumulation and bacterial load by modulating key cholesterol-related genes. These effects were confirmed in an *in vivo* murine mastitis model, where fenofibrate alleviated tissue inflammation, normalized autophagy-lysosomal function, and significantly decreased pathogen burden. This study highlighted the potential of fenofibrate as an effective therapeutic approach for intracellular *M. bovis* infection, modulating autophagy and lipid metabolism (**Figure 10**).

## Data Availability

All data generated or analyzed during this study are included in this published article.
